# Development and Characterization of a Novel Erucyl Ultra-Long-Chain Gemini Surfactant

**DOI:** 10.3390/polym17162257

**Published:** 2025-08-21

**Authors:** Guiqiang Fei, Banghua Liu

**Affiliations:** College of Chemistry and Chemical Engineering, Shaanxi University of Science & Technology, Xi’an 710021, China; bs240811025@sust.edu.cn

**Keywords:** gemini surfactant, erucyl, ultra-long chain, surface activity, viscoelasticity

## Abstract

To stimulate the progress of clean fracturing fluid systems, an innovative erucyl ultra-long-chain gemini surfactant (EUCGS) was devised and manufactured during the course of this study. The target product was successfully prepared via a two-step reaction involving erucyl primary amine, 3-bromopropionyl chloride, and 1,3-bis(dimethylamino)propanediol, with an overall yield of 78.6%. FT-IR and ^1^H NMR characterization confirmed the presence of C_22_ ultra-long chains, cis double bonds, amide bonds, and quaternary ammonium headgroups in the product structure. Performance tests showed that EUCGS exhibited an extremely low critical micelle concentration (CMC = 0.018 mmol/L) and excellent ability to reduce surface tension (γCMC = 30.0 mN/m). Rheological property studies indicated that EUCGS solutions gradually exhibited significant non-Newtonian fluid characteristics with increasing concentration, and wormlike micelles with a network structure could self-assemble at a concentration of 1.0 mmol/L. Dynamic rheological tests revealed that the solutions showed typical Maxwell fluid behavior and significant shear-thinning properties, which originated from the orientation and disruption of the wormlike micelle network structure under shear stress. In the presence of 225 mmol/L NaCl, the apparent viscosity of a 20 mmol/L EUCGS solution increased from 86 mPa·s to 256 mPa·s. A temperature resistance evaluation showed that EUCGS solutions had a good temperature resistance at high shear rates and 100 °C. The performance evaluation of fracturing fluids indicates that the proppant settling rate (0.25 cm/min) of the EUCGS-FFS system at 90 °C is significantly superior to that of the conventional system. It features the low dosage and high efficiency of the breaker, with the final core damage rate being only 0.9%. The results demonstrate that the EUCGS achieves a synergistic optimization of high-efficiency interfacial activity, controllable rheological properties, and excellent thermal–salt stability through precise molecular structure design, providing a new material choice for the development of intelligent responsive clean fracturing fluids.

## 1. Introduction

With stable progress in the exploration and utilization of unconventional oil and gas resources, hydraulic fracturing technology has emerged as a leading stimulation approach, experiencing widespread deployment in different sectors [1]. Traditional fracturing fluid systems suffer from numerous issues such as environmental pollution and reservoir damage, making the development of efficient and eco-friendly clean fracturing fluids a current research hotspot [2,3]. As the core component of clean fracturing fluids, surfactants exhibit a close relationship between their molecular structure and performance [4].

Gemini surfactants, a special class of surfactants composed of two hydrophilic headgroups and two hydrophobic groups linked by a spacer group, possess unique advantages over traditional surfactants, including low critical micelle concentration (CMC), strong surface tension reduction ability, and diverse self-assembly behaviors [5,6,7]. Bhut et al. [8] demonstrated that gemini surfactants can form complex network micelle structures at low concentrations, exhibiting excellent viscoelasticity—a property crucial for the proppant-carrying capacity of fracturing fluids. Roshdy et al. [9] found that cationic gemini surfactants possess good temperature stability and interfacial activity, maintaining superior performance under high-temperature and high-pressure conditions.

Erucyl-based surfactants, characterized by their long carbon chain (C22) and cis double bonds, exhibit a unique tortuous configuration in their molecular structure, leading to distinctive self-assembly properties [10]. Moore et al. [11] reported that surfactants derived from erucyl amine can form stable wormlike micelles, demonstrating significant viscoelasticity and shear-thinning behavior. However, most current erucyl-based surfactants have a single-chain structure, and their stability and performance regulation flexibility under extreme conditions still need improvement [12].

Combining the erucyl structure with the molecular design concept of gemini surfactants holds promise for developing novel surfactants with superior performance [13]. Theoretically, the ultra-long-chain gemini structure can significantly enhance intermolecular hydrophobic interactions, reduce the critical micelle concentration, and improve micelle stability [14]. Zhang et al. [15] independently synthesized two types of cationic surfactants featuring erucyl amide moieties and varying spacer groups. Despite their excellent performance, these surfactants exhibit poor temperature tolerance. Han et al. [16] revealed through molecular dynamics simulations that increasing the hydrophobic chain length in amphiphilic molecules significantly affects their self-assembly behavior and rheological properties. However, systematic research on erucyl ultra-long-chain gemini surfactants remains scarce.

In this study, a novel erucyl ultra-long-chain gemini surfactant was designed and synthesized using erucyl primary amine, 3-bromopropionyl chloride, and 1,3-bis(dimethylamino)propanediol as the main raw materials. To determine the structure of the product, ^1^H NMR and FT-IR techniques were applied, and key performance indicators including surface activity, rheological properties, pH responsiveness, and temperature resistance were systematically evaluated, providing new high-efficiency functional materials for the clean fracturing fluid field.

## 2. Experimental Materials and Methods

### 2.1. Experimental Materials

Erucyl amine (EA, purity ≥ 98%) and bromopropionyl chloride (purity ≥ 97%) were purchased from Jiangsu Rain Environmental Protection Technology Co., Ltd. (Nantong, China). 1,3-bis(dimethylamino)propan-2-ol (purity ≥ 98%), triethylamine (analytical grade), anhydrous calcium chloride (analytical grade), tetrahydrofuran (chromatographic grade), methanol (chromatographic grade), ether, acetone, and chloroform were all obtained from Nantong Runfeng Petrochemical Co., Ltd. (Nantong, China). Hydrochloric acid within the concentration of 36–38% as well as analytical-grade sodium hydroxide were acquired from Beijing Chemical Plant (Beijing, China). Deionized water was used in the experiments. All reagents were used without further purification.

### 2.2. Experimental Instruments

For the experiment, the subsequent instruments were employed: an AVANCE III HD 400 MHz nuclear magnetic resonance spectrometer purchased from Bruker (Karlsruhe, Germany); a Nicolet iS50 Fourier transform infrared spectrometer made by Thermo Fisher (Los Angeles, CA, USA); a Dingsheng JYW-200A surface tension meter (Chengde Yote Instrument Manufacturing Co., Ltd., Chende, China); a Discovery HR-2 rheometer provided by TA Instruments (New Castle, DE, USA); a PHS-3C pH meter from Shanghai Yidian Sci. Instrument Co., Ltd. (Shanghai, China); an MS-H-S heating magnetic stirrer from DLAB Sci. Instruments Co., Ltd. (Beijing, China); an RE-52AA rotary evaporator from Shanghai Yarong Biochem. Instrument Factory (Shanghai, China); an FD-1A-50 vacuum freeze dryer supplied by Beijing Boyikang Exp. Instrument Co., Ltd. (Beijing, China); an AL204 electronic balance from Mettler-Toledo Shanghai (Shanghai, China); a KQ-500DE digital ultrasonic cleaner from Kunshan Ultrason. Instruments Co., Ltd. (Kunshan, China); a DHG-9070A electric heating constant temperature blast drying oven from Shanghai Yiheng Sci. Instrument Co., Ltd. (Shanghai, China); and an SHZ-D (III) circulating water multi-purpose vacuum pump from Zhengzhou Great Wall Sci. & Ind. Co., Ltd. (Zhengzhou, China).

### 2.3. Preparation of Erucyl Ultra-Long-Chain Gemini Surfactant

**Figure 1:**
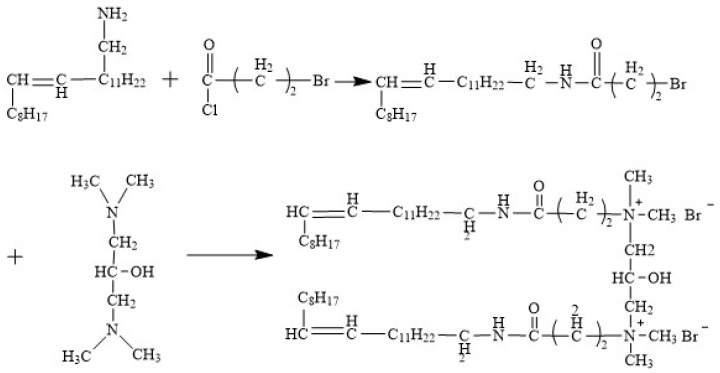
The synthetic pathway of EUCGS.

The synthetic route of the erucyl-based ultra-long-chain gemini surfactant (EUCGS) is shown in Figure 1, which mainly involves two-step reactions.

#### 2.3.1. Synthesis of Erucyl Bromopropionamide Intermediate

A total of 10.0 g (30 millimoles) of erucyl primary amine and 60 milliliters of tetrahydrofuran were placed into a 250 mL three-necked flask. Stirring was carried out until the erucyl primary amine was thoroughly dissolved in the tetrahydrofuran solvent. An ice–salt bath was employed to cool the reaction system to 0 °C. Subsequently, 6.06 g (60 mmol) of triethylamine was added in a slow, dropwise fashion. During continuous agitation, 7.64 g (45 millimoles) of bromopropionyl chloride in 20 milliliters of tetrahydrofuran solution was slowly introduced using a constant pressure dropping funnel, and the addition process lasted for one hour. After the addition step was fully accomplished, the reaction mixture underwent agitation at 0 °C for two hours. Following this, the temperature of the mixture was adjusted to room temperature, and the reaction was sustained for an additional 24-h interval.

After the reaction, triethylamine hydrochloride precipitate was filtered off. Under reduced pressure conditions, the filtrate was concentrated to expel the majority of tetrahydrofuran. Then, 50 mL of ether was incorporated into the concentrated filtrate, and the formed mixture was subsequently transferred into a separatory funnel for subsequent separation procedures. The mixture underwent a series of washing steps. To start with, the object under study was washed thrice with 20 mL of a 5% hydrochloric acid solution, and then it was further washed three times with 20 mL of a 5% sodium carbonate solution. Finally, the mixture was rinsed three times with 30 mL of saturated brine. Anhydrous sodium sulfate was employed to dry the organic phase throughout the night. Upon finishing the drying process, filtration was carried out on the mixture, and the solvent was subsequently removed via a rotary evaporator, leading to the obtaining of a light-yellow solid product. The solid was recrystallized from an ice-cold methanol/ether (1:4, *v/v*) mixture, filtered, and dried to yield the white solid erucyl bromopropionamide intermediate (abbreviated as BrPA). The calculated yield was determined to be 86.2%. 

#### 2.3.2. Synthesis of Erucyl Ultra-Long-Chain Gemini Surfactant

In a 250 mL three-necked flask, 1.46 g (10 mmol) of 1,3-bis(dimethylamino)propan-2-ol and 50 mL of anhydrous tetrahydrofuran were added and stirred until completely dissolved. A total of 6.8 g (25 mmol) of Product 1 (BrPA) was carefully incorporated into the reaction setup. The resultant mixture underwent stirring at room temperature conditions for 30 min. Afterwards, the temperature was raised to 60 °C, and the reaction was conducted over a period of 48 h. Periodic evaluations of the reaction’s development were performed using thin-layer chromatography.

After the reaction reached its completion, the reaction mixture was cooled to ambient conditions, and then the solvent was eliminated by means of rotary evaporation. The residue was solubilized in a minimal amount of chloroform. Afterward, an overabundance of acetone was added to precipitate the components. The suspension was subjected to filtration, after which the solid precipitate obtained was washed three times with acetone. Finally, vacuum drying was carried out, leading to the acquisition of the white powdery erucyl ultra-long-chain gemini surfactant, denoted as EUCGS for brevity. The calculated yield was determined to be 91.2%.

### 2.4. Structure Characterization and Performance Evaluation Methods

#### 2.4.1. Structure Characterization

For the FT-IR analysis, a minuscule quantity of the sample was homogeneously blended and ground with KBr. Subsequently, the mixture was compressed into a pellet and then subjected to scanning on a Nicolet iS50 Fourier transform infrared spectrometer (Thermo Fisher Scientific, Los Angeles, CA, USA). Scanning operations were implemented in the wavenumber spectrum spanning from 400 to 4000 cm^−1^, with an analytical resolution set at 4 cm^−1^. Moreover, 32 individual scans were completed.

For the ^1^H NMR characterization, 5 to 10 milligrams of the specimen were dissolved in 0.6 milliliters of deuterated chloroform (CDCl_3_). The hydrogen nuclear magnetic resonance (1H NMR) spectral data were collected on an AVANCE III HD 400 MHz spectrometer(Bruker, Karlsruhe, Germany). Tetramethylsilane was applied as the internal standard to ensure accurate spectral analysis. All the spectral data were collected under ambient temperature conditions.

#### 2.4.2. Surface Activity Evaluation

Critical Micelle Concentration (CMC) Measurement: The CMC of EUCGS was determined via the surface tension method. EUCGS aqueous solutions with various concentrations ranging from 0.001 to 1.000 mmol/L were formulated, and their surface tension values were measured at 25 °C using a JYW-200A surface tension meter (TA Instruments, New Castle, DE, USA). A graphical illustration was created to show the variation in surface tension with respect to the logarithmic concentration values, and the CMC was determined as the concentration value at which the inflection point of the curve occurred. The number of measurements was 3, with a relative standard deviation of less than 2%.

Calculation of Surface Adsorption Parameters: The Gibbs adsorption isotherm equation was employed to determine the maximum surface excess concentration (Γmax) and the minimum molecular occupied area (Amin) of EUCGS at the gas/liquid interface. 

#### 2.4.3. Rheological Property Evaluation

Steady-state rheological measurements: A Discovery HR-2 rheometer(TA Instruments, New Castle, DE, USA) equipped with a coaxial cylinder measurement system (inner cylinder diameter: 26.0 mm, gap size: 1.0 mm) was employed. Aqueous solutions of EUCGS with concentrations of 0.5, 1, 5, 10, 15, and 20 mmol/L were prepared and subjected to continuous testing at 25 °C over a shear rate range from 0.01 s^−1^ to 1000 s^−1^, aiming to obtain the relationship curves between the apparent viscosity and shear rate.

Dynamic rheological measurements: Using the same rheometer, frequency sweep tests were conducted on 1, 5, 10, 15, and 20 mmol/L EUCGS aqueous solutions within an angular frequency range of 0.01 to 100 rad/s. The strain was controlled within the linear viscoelastic region to acquire the relationship curves of the storage modulus (G’) and loss modulus (G”) with angular frequency. 

#### 2.4.4. Salt Effect Study

The impact of varying concentrations of sodium chloride (NaCl) and calcium chloride (CaCl_2_) on the rheological characteristics of EUCGS solutions was explored under conditions of 25 °C temperature and a shear rate of 170 rad/s. 

#### 2.4.5. Temperature Resistance Evaluation

Multiple sealed test tubes were filled with a 1.0 mmol/L EUCGS aqueous solution, and subsequent rheological measurements were performed at six distinct temperatures (25 °C, 50 °C, 75 °C, 100 °C, 125 °C, and 150 °C) under a fixed shear rate of 170 rad/s. To ensure an accurate temperature equilibration, each sample was maintained at the target temperature for 15 min prior to measurement, with the temperature fluctuation strictly controlled within ±1 °C. These experiments were specifically designed to systematically evaluate how temperature influences the stability of EUCGS through the analysis of rheological properties.

### 2.5. Performance Evaluation of Fracturing Fluid

The developed EUCGS was used to prepare a fracturing fluid system, which was compared with the fracturing fluid system prepared by traditional cationic surfactants in terms of performance. The specific performance evaluations included the proppant-carrying performance evaluation, gel-breaking performance evaluation, and core damage performance evaluation.

The fracturing fluid system composed of EUCGS (EUCGS-FFS) was formulated as follows: 0.4% EUCGS + 0.3% KCl. The fracturing fluid system composed of Cetyltrimethylammonium bromide (CTAB) (CTAB-FFS) was formulated as follows: 0.4% CTAB + 0.3% KCl.

#### 2.5.1. Evaluation of Proppant-Carrying Performance

After heating the fracturing fluid sample to the preset temperature, ceramic proppants with a mass fraction of 10% and a particle size range of 0.425–0.850 mm were incorporated, and their autonomous settling process was observed. By measuring the time required for the ceramic proppants to settle a specific distance, the settling velocity was calculated, thereby evaluating the sand-carrying performance of the target fracturing fluid.

#### 2.5.2. Evaluation of Gel-Breaking Performance

A total of 100 mL of the target fracturing fluid was placed in a beaker and heated to the preset temperature using a water bath. A certain volume of kerosene or absolute ethanol was added under stirring conditions, followed by static treatment in a 50 °C environment. After standing for 2 h, the upper clear liquid was taken for viscosity measurement. Complete gel breaking was determined when the solution viscosity dropped to 5 mPa·s, and the time required to reach this state was recorded as the gel-breaking time.

#### 2.5.3. Evaluation of Core Damage Performance

**Figure 2:**
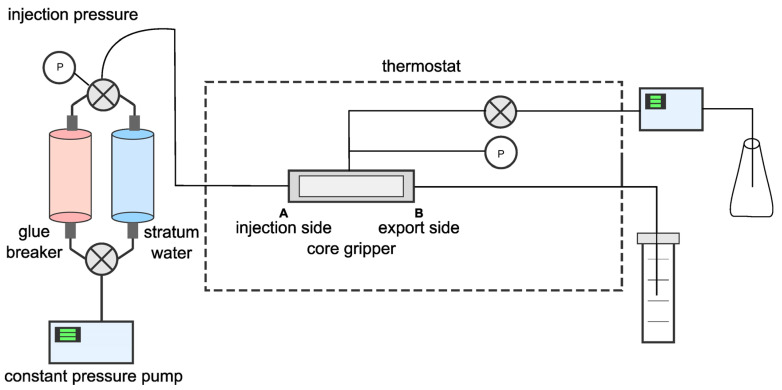
Diagram of core damage experimental device.

The artificial core was first weighed for dry weight and recorded as *m*_1_. Subsequently, it was placed in a core holder (see Figure 2), and the confining pressure was maintained at 5 MPa.

Standard brine was injected into the artificial core at a flow rate of 1 mL/min. Meanwhile, the pressure variation process was recorded. After the pressure tended to be stable, the injection pressure value Δ*P*_0_ was read. Then, the core was weighed again and recorded as *m*_2_. The initial permeability *K*_0_ was calculated by the following formula.

(1) $$K=\frac{Q\mu L}{10A\Delta P}$$

In the equation:

*K* denotes the core permeability, with the unit of mD (millidarcy);

*Q* represents the fluid injection rate, in the unit of cm^3^/s;

*μ* refers to the viscosity of the injected fluid, measured in mPa·s;

*L* signifies the core length, with the unit of cm;

*A* is the cross-sectional area of the core, in the unit of cm^2^;

Δ*P* indicates the pressure difference across the two ends of the core, measured in MPa.

Continue to inject 1.3 Pore Volume (PV) of gel-breaking fluid into the core at a flow rate of 1 mL/min; record the pressure changes in real time. After the pressure stabilizes, read the injection pressure value Δ*P*_1_, thereby calculating the core permeability *K*_1_, and measure the core permeability damage rate Φ according to the following formula:

(2) $$\Phi =\frac{K_{0}-K_{1}}{K_{0}}\times 100\%$$

Inject 2.5 PV of standard brine at a constant flow rate of 1 mL/min; record the pressure variation throughout the process. After the pressure stabilizes, read the injection pressure value Δ*P*_2_. Calculate the core permeability recovery ratio *R* and the final core damage rate Φ′ by using Equation (3) and Equation (4), respectively.

(3) $$R=\frac{K_{2}-K_{1}}{K_{1}}\times 100\%$$

(4) $$\Phi^{\prime }=\frac{K_{0}-K_{2}}{K_{0}}\times 100\%$$

## 3. Results and Discussion

### 3.1. Structural Characterization

#### 3.1.1. FT-IR Characterization

**Figure 3:**
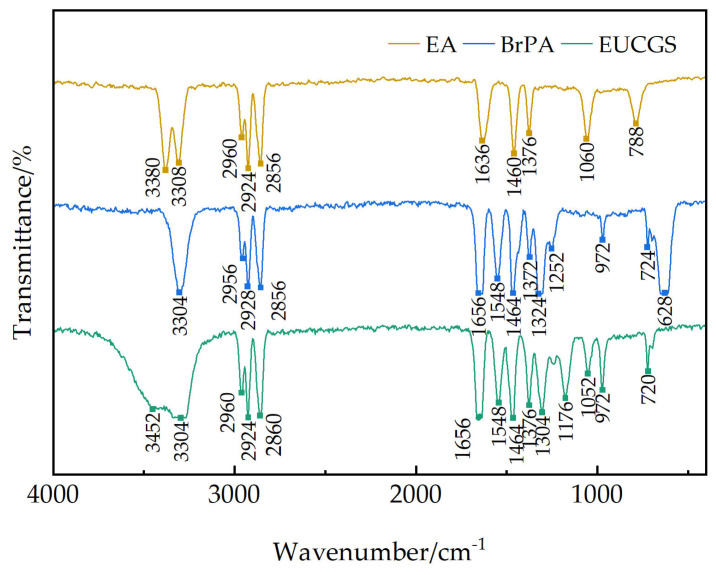
Infrared spectrogram.

Figure 3 shows the FT-IR spectra of erucyl primary amine, erucyl bromopropionamide intermediate, and the final product EUCGS. In the infrared spectrum of erucyl primary amine, the absorption peaks at 3380 cm^−1^ and 3308 cm^−1^ correspond to the asymmetric and symmetric stretching vibrations of NH_2_, respectively. A series of characteristic CH_3_ and CH_2_ stretching vibration peaks appear in the region of 2960–2855 cm^−1^ [17], among which the strong absorption at 2924 cm^−1^ reflects the presence of the long-chain structure. The medium-intensity absorption at 1636 cm^−1^ is attributed to the stretching vibration of the C=C double bond.

In the infrared spectrum of the erucyl bromopropionamide intermediate, a strong amide N-H stretching vibration appears at 3304 cm^−1^ [18], and a typical amide I band (C=O stretching) is observed at 1656 cm^−1^ [19]. The characteristic C-Br stretching vibrations are clearly visible at 640 cm^−1^ and 620 cm^−1^ [20]. Notably, the characteristic absorption of α-bromoamide appears at 1324 cm^−1^ [21], while the original NH_2_ characteristic peaks (3380 and 3308 cm^−1^) disappear, confirming the completion of the amidation reaction.

In the infrared spectrum of EUCGS, a broad peak of hydroxyl (-OH) appears at 3420 cm^−1^. The C-O stretching vibration is clearly visible at 1176 cm^−1^, and the C-N stretching vibration at 1304 cm^−1^ confirms the formation of the quaternary ammonium structure [22]. The disappearance of the C-Br characteristic peaks at 640 and 620 cm^−1^ indicates the complete substitution of the bromo group. Additionally, the strong amide C=O stretching vibration retained at 1656 cm^−1^ and the amide II band at 1548 cm^−1^ suggest that the amide structure remains intact.

Through comparative analysis of the FT-IR spectra, it can be confirmed that the structures of the erucyl bromopropionamide intermediate and the final product EUCGS are consistent with the design, indicating the successful realization of the synthetic route.

#### 3.1.2. ^1^H NMR Characterization

**Figure 4:**
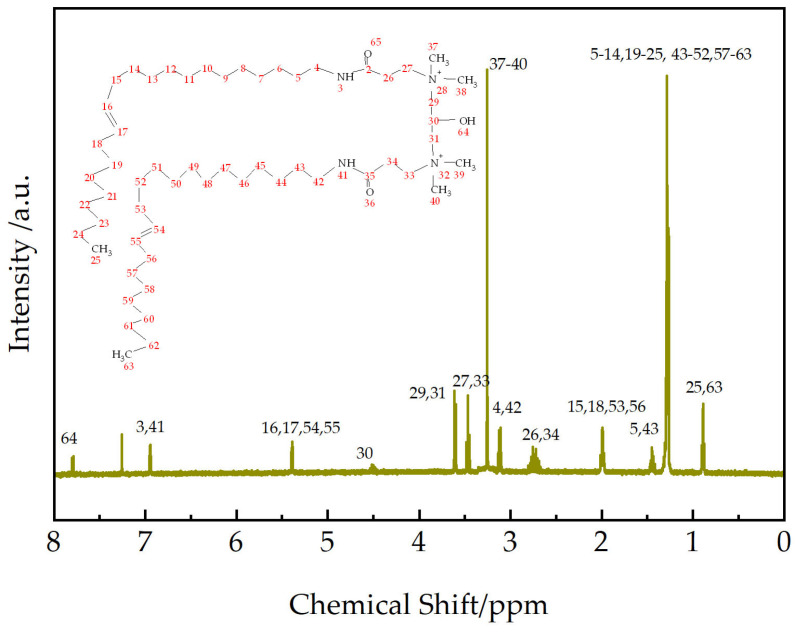
^1^H NMR spectrum (CDCl_3_, 25 °C) of EUCGS.

To further confirm the product structure, ^1^H NMR spectroscopy analysis was performed on EUCGS, and the results are shown in Figure 4.

The ^1^H NMR spectrum of EUCGS (as shown in Figure 4) provides definitive evidence for the product structure. The main characteristic chemical shifts (δ, ppm) and their assignments are as follows: 0.89 (-CH_2_-CH_3_, terminal methyl); 1.26–1.31 (-(CH_2_)_n_-, long-chain methylene); 1.45 (-CH_2_-CH_2_-NH-, β-methylene hydrogen); 1.98–2.02 (-CH_2_-CH=CH-CH_2_-, allylic methylene); 2.70–2.78 (-CO-CH_2_-CH_2_-N<, methylene at α-position of carbonyl); 3.12 (-CH_2_-CH_2_-NH-, α-methylene hydrogen); 3.25 (>N^+^(CH_3_)_2_, quaternary ammonium methyl); 3.46 (-CO-CH_2_-CH_2_-N<, methylene at α-position of nitrogen); 3.61 (>N-CH_2_-CH<, methylene in connecting group); 4.51 (>CH-OH, hydroxyl methine); 5.39 (-CH=CH-, olefinic hydrogen) [23]; 6.95 (-NH-CO-, amide hydrogen); and the peak at 7.25 is attributed to the residual proton signal in the CDCl_3_ solvent.

Among them, the distinct signal of quaternary ammonium methyl hydrogen at δ = 3.25 ppm and the signal assigned to methylene hydrogen in the connecting group at δ = 3.61 ppm [24] strongly confirm the successful introduction of the connecting group and the formation of the EUCGS molecular skeleton.

### 3.2. Performance Evaluation

#### 3.2.1. Surface Activity Evaluation

**Figure 5:**
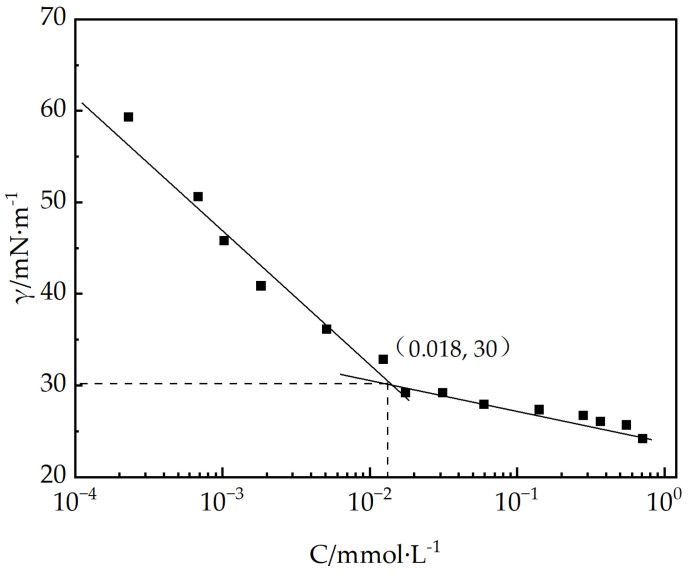
The surface tension diagram of EUCGS.

By spontaneously adsorbing at the gas/liquid interface, surfactant molecules lower the free energy of the system, and there exists a close relationship between the surface activity of these molecules and their molecular configuration. Figure 5 demonstrates the association between the surface tension (γ) and the concentration (C) of EUCGS aqueous solutions measured at 25 °C. As the concentration of EUCGS rises, there is a rapid decrease in the surface tension of the solution, and it tends to stabilize when the concentration reaches a critical value. The CMC of EUCGS was determined to be 0.013 mmol/L by the inflection point of the curve, with a corresponding surface tension value (γ_CMC_) of 30 mN/m.

For comparison, Table 1 presents the surface activity parameters of the EUCGS and several typical surfactants reported in the literature. The CMC value of EUCGS is significantly lower than that of the traditional single-chain surfactant cetyltrimethylammonium chloride (CTAAC, approximately 0.15 mmol/L), as well as general gemini surfactants JS-N-JS (0.028 mmol/L) and VES-Q (0.047 mmol/L) [25]. This result indicates that the synergistic effect of the erucyl ultra-long-chain structure and the gemini molecular design endows EUCGS with an extremely low CMC. According to the research by Menger and Keiper [14], a lower CMC value implies a stronger self-aggregation ability and higher efficiency of surfactant molecules, which is of great significance for reducing the dosage in practical applications, lowering costs, and minimizing environmental burdens.

**Figure 6:**
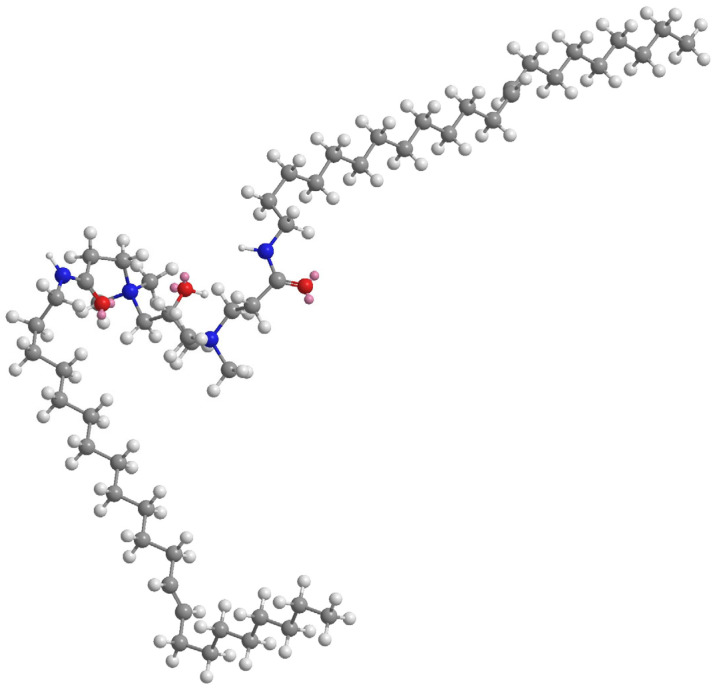
Three-dimensional structure of EUCGS (generated using Chem3D v16.0 software with the calculations tool and GAMESS interface module).

Calculated from the Gibbs adsorption isotherm equation, the Γmax of EUCGS at the gas/liquid interface is 1.28 × 10^−3^ mmol/cm^2^, with a corresponding minimum molecular occupied area (Amin) of 1.30 nm^2^. Compared with traditional single-chain surfactants, EUCGS has a larger Amin value, which is consistent with its molecular structural characteristics. Wei et al. pointed out that due to their special molecular configuration, gemini surfactants occupy a larger area during interfacial arrangement. The cis double bond structure in the erucyl chain of EUCGS causes the molecular chain to exhibit a bent configuration, further increasing the molecular occupied area at the interface (as shown in Figure 6).

In summary, the EUCGS exhibits outstanding surface activity. The remarkably low CMC value and the superior surface tension decreasing performance of this substance result from the combined action of the erucyl ultra-long-chain segment and the gemini structural motif present in its molecular framework. These distinctive properties render the EUCGS an ideal candidate for surfactants in the field of clean fracturing fluids.

#### 3.2.2. Evaluation of Rheological Properties

Surfactant molecules in aqueous solutions undergo self-assembly to form diverse aggregates, including spherical micelles, rod-like micelles, and wormlike micelles. The structures of these aggregates are intricately associated with factors such as concentration, temperature, and the presence of additives. Concurrently, these structural characteristics dictate the rheological properties of the solution. In the context of hydraulic fracturing fluid applications, surfactant solutions must exhibit appropriate viscoelastic properties to ensure effective proppant transport capabilities and drag reduction performance [29].

(1) Steady-State Rheology

**Figure 7:**
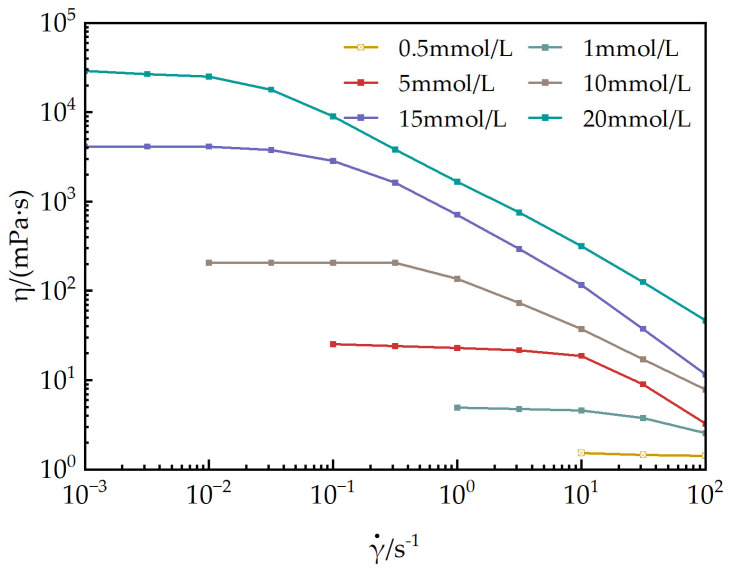
Steady-state rheology of EUCGS.

Figure 7 illustrates the relationship between the apparent viscosity and shear rate of EUCGS aqueous solutions at various concentrations under a constant temperature of 25 °C. When the concentration is below 0.5 mmol/L, the solutions exhibit predominantly Newtonian fluid behavior, with the apparent viscosity remaining nearly constant regardless of changes in the shear rate. Conversely, when the concentration reaches 1.0 mmol/L, the solutions display significant non-Newtonian characteristics, specifically shear-thinning behavior.

According to the research of Dreiss [30], the shear-thinning behavior of the surfactant solution mainly stems from the orientation and disruption of the wormlike micelle network structure under shear action. The erucyl (EUCGS) ultra-long-chain structure promotes strong intermolecular hydrophobic interactions, which are conducive to the formation of wormlike micelles with good flexibility. This rheological property is of great significance for fracturing fluids: It has a relatively high viscosity under low shear conditions (in a static state or at the initial stage of pumping), which is beneficial for the suspension and transportation of proppants; its viscosity decreases under high shear conditions (when passing through pumps and pipelines), reducing energy consumption; and the viscosity can be restored after the shear is eliminated, ensuring the sand-carrying capacity after reaching the fractures [31].

(2) Dynamic Rheology

**Figure 8:**
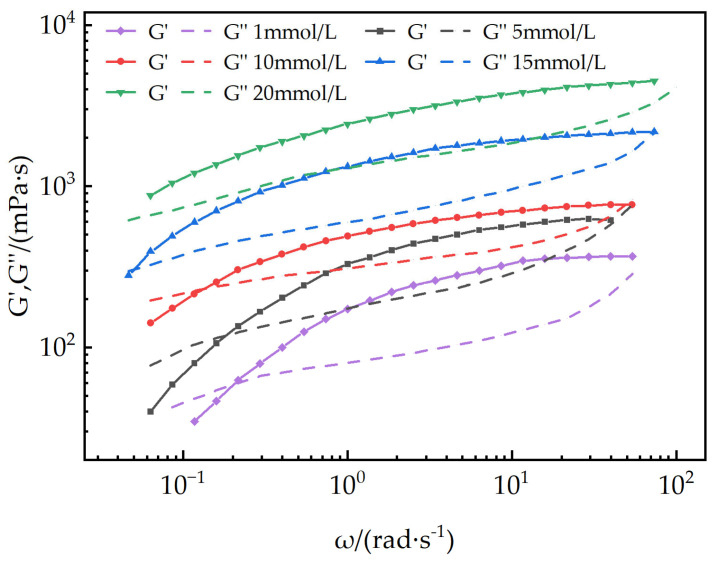
Dynamic rheology of EUCGS.

To further investigate the viscoelastic characteristics of the EUCGS solution, a dynamic oscillatory rheological test was conducted. Figure 8 presents a comparison of the dynamic modulus spectra of EUCGS solutions with different concentrations. In the frequency range of ω < 0.1 rad/s, which is characterized as the low-frequency domain, G’’ exceeds G’. This observation indicates that the solution predominantly demonstrates a viscous behavior. As the frequency increases, the growth rate of G’ is faster than that of G’’, and the two become equal at the crossover frequency. When operating at high frequencies, G’ surpasses G’’, indicating that the solution displays a behavior mainly governed by elasticity. As the concentration rises, there is a notable augmentation in both G’ and G’’. Additionally, the crossover frequency ω shifts towards the low-frequency region. The reciprocal of ω_c_ can be used to estimate the relaxation time τ_R_ of the system. As can be observed from Figure 7, when the solution concentration increases from 5.0 mmol/L to 15 mmol/L, the relaxation time continuously increases, indicating that under high-concentration conditions, the micellar network structure becomes denser and more stable, and the elasticity of the system is enhanced.

#### 3.2.3. Result on the Salt Effect

**Figure 9:**
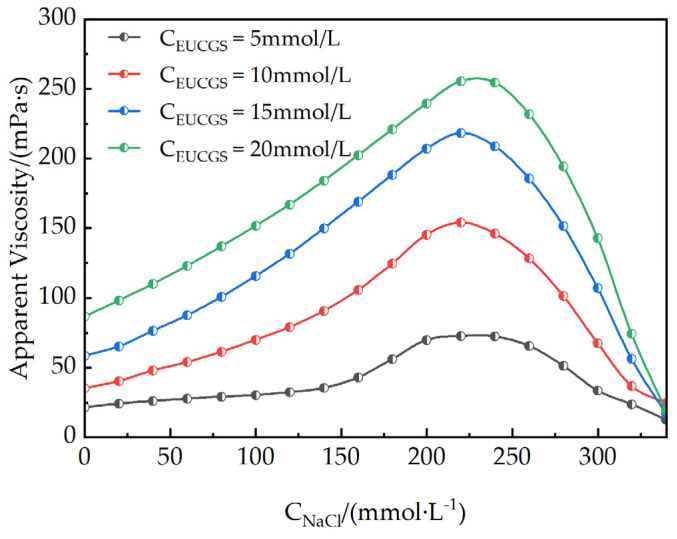
Apparent viscosity of EUCGS under different NaCl concentrations.

**Figure 10:**
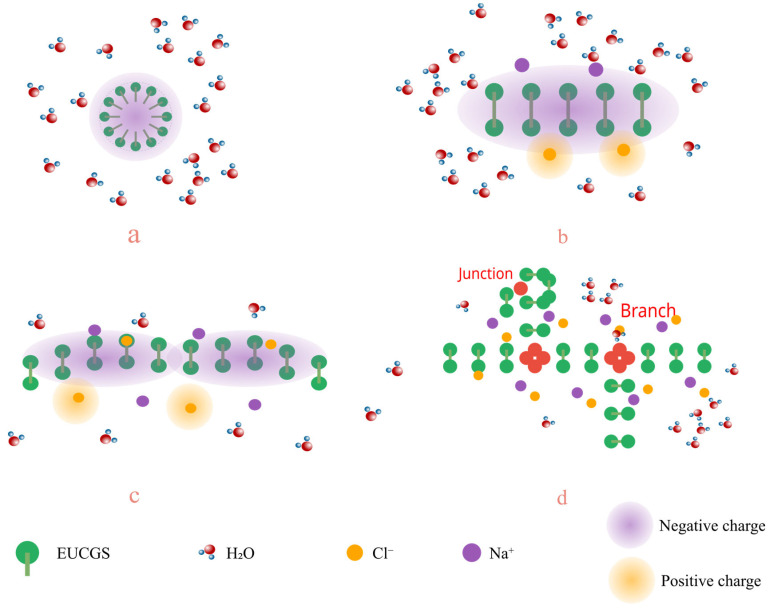
Schematic diagram of the mechanism of action of the salt effect of EUCGS (**a**) low salt concentration; (**b**) low to medium salt concentration; (**c**) medium salt concentration; (**d**) high salt concentration.

The addition of electrolytes can significantly affect the self-assembly behavior and rheological properties of surfactants. The impact of varying NaCl concentrations on the rheological characteristics of EUCGS solutions at different concentrations is depicted in Figure 9. As the NaCl concentration is incremented to 225 mmol/L, the solution exhibits its highest apparent viscosity. Regarding the impact of EUCGS on the apparent viscosity, distinct increments are observed at various concentrations. When the EUCGS concentration stands at 5 mmol/L, there is an increase in the apparent viscosity from 21 mPa·s to 73 mPa·s. At 10 mmol/L of EUCGS, the value of the apparent viscosity advances from 35 mPa·s to 154 mPa·s. When the EUCGS concentration amounts to 15 mmol/L, the apparent viscosity progresses from 58 mPa·s to 218 mPa·s, and at 20 mmol/L, it progresses from 86 mPa·s to 256 mPa·s. The primary reason for this phenomenon is that salt ions neutralize the electrostatic repulsion among the head groups of EUCGS molecules, as illustrated in Figure 10b,c. Upon neutralization, there is a decrease in the available area of the head groups, and this change subsequently supports the elongation of micellar structures and the generation of wormlike morphologies. Nevertheless, upon further elevation of the NaCl concentration to 250 mmol/L, a reduction in the apparent viscosity is observed rather than an increase [32]. As is shown in Figure 10d, according to previous studies [33,34], this viscosity decrease is attributed to a transition from linear to branched micelles when the electrostatic repulsion is sufficiently screened by the high ionic strength. At high NaCl concentrations, the reduced electrostatic repulsion lowers the end-cap energy of the micelles, making branch point formation thermodynamically favorable, which introduces additional stress relaxation mechanisms and consequently reduces the solution viscosity [33].

**Figure 11:**
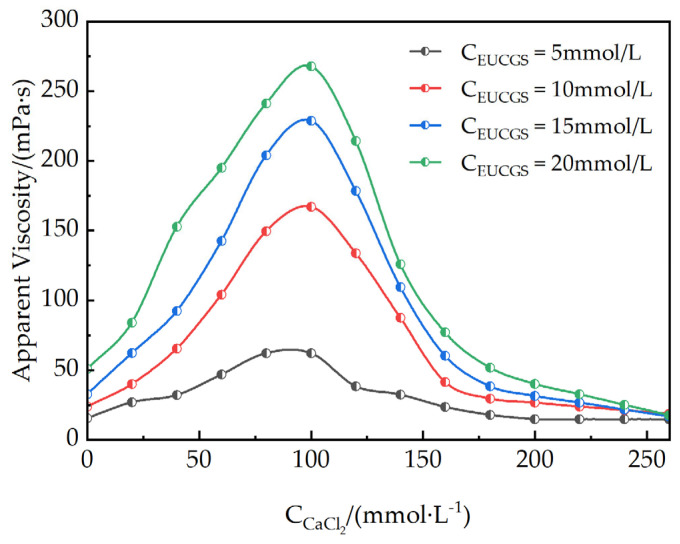
Apparent viscosity of EUCGS under different CaCl_2_ concentrations.

Figure 11 illustrates the apparent viscosity curves of EUCGS at different concentrations in CaCl_2_ solutions of various concentrations. The graphical representation clearly demonstrates that the effect of CaCl_2_ on the rheological behavior of EUCGS solutions presents both similarities and differences when compared to the influence of NaCl. In the starting period of the step-by-step increments of CaCl_2_ concentration, the apparent viscosity of the EUCGS solution rises remarkably. Contrasting with NaCl, Ca^2+^, in its capacity as a divalent cation, demonstrates a more effective shielding property against the electrostatic repulsion of the head groups of EUCGS molecules. This stronger shielding effect enables Ca^2+^ to more effectively reduce the effective head group area, facilitating a more rapid transformation of micelles into wormlike structures, thereby substantially enhancing the solution viscosity. Upon the CaCl_2_ concentration reaching around 100 mmol/L, the apparent viscosity of the 5 mmol/L EUCGS solution experiences a significant elevation, surging from the initial value of 15.41 mPa·s to 62.2 mPa·s. This increment is substantially greater than that induced by NaCl at an equivalent concentration. In addition, for the EUCGS solution at a concentration of 10 mmol/L, the apparent viscosity escalates sharply from 23.60 mPa·s to 167.22 mPa·s. Such a dramatic rise provides compelling evidence for the enhanced promoting effect of divalent cations.

However, when the concentration of CaCl_2_ continues to increase and exceeds 150 mmol/L, the apparent viscosity of the solution does not keep rising but drops rapidly. This phenomenon differs from the NaCl system and can be attributed to the formation of branched wormlike micelles induced by the high ionic strength. The high concentration of Ca^2+^ effectively screens the electrostatic repulsion between surfactant headgroups, reducing the end-cap energy and promoting branch point formation. When the branch point energy becomes lower than twice the end-cap energy (E_b_ < 2E_c_), the linear micelles transform into branched structures, introducing additional stress relaxation pathways that significantly reduce the solution viscosity while maintaining optical clarity.

The divalent nature of Ca^2+^ makes it more efficient than Na^+^ in inducing this branching transition. Additionally, Ca^2+^ may interact with specific groups in the EUCGS molecules through weak complexation, which could further modulate the micellar packing parameter and facilitate branch formation [35,36]. This multi-modal interaction mechanism, involving both enhanced electrostatic screening and specific ion–head group interactions, explains why the CaCl_2_ system exhibits a more pronounced viscosity reduction compared to the NaCl system at equivalent ionic strengths.

It should be noted that at very high CaCl_2_ concentrations in dilute EUCGS systems, solution stratification may occur, indicating a transition to phase separation under these extreme conditions. This observation suggests that the Ca^2+^-EUCGS interaction follows a concentration-dependent pathway: from linear micelle growth at low salt concentrations to branched micelle formation at intermediate concentrations and potentially to phase separation at very high salt concentrations when the surfactant concentration is low.

#### 3.2.4. Evaluation of Temperature Resistance

**Figure 12:**
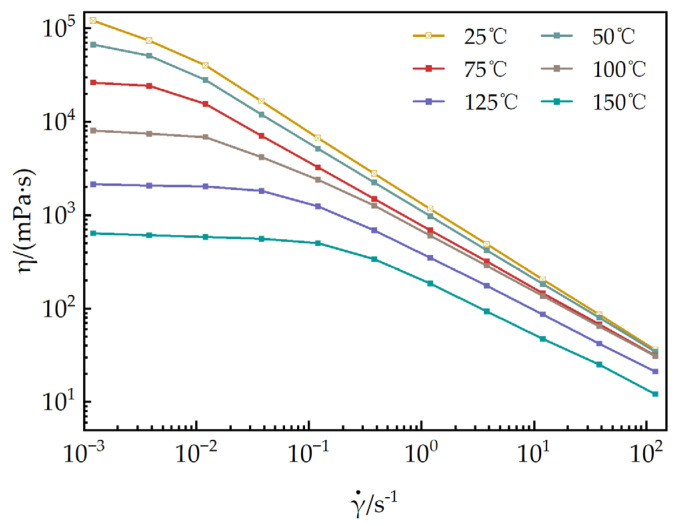
Impact of temperature on the steady-state rheological behaviors of a 1 mmol/L EUCGS solution.

**Figure 13:**
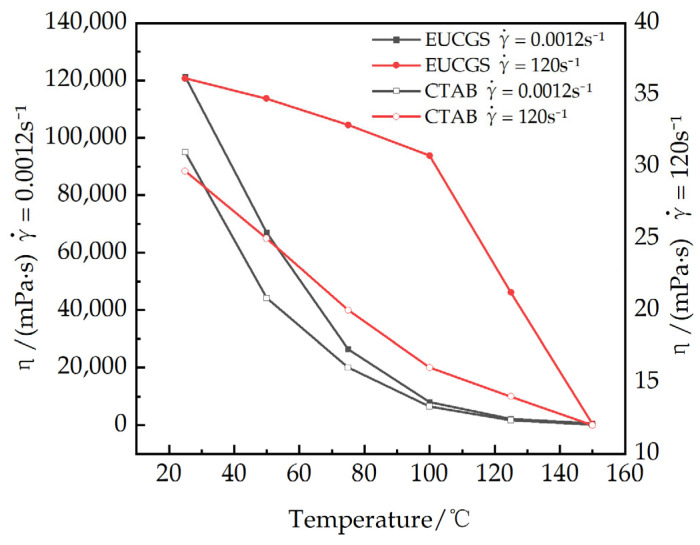
Viscosity of EUCGS and CTAB Variation with Temperature under Different Shear Rates.

As shown in Figure 12, the steady-state rheological behaviors of a 1 mmol/L EUCGS solution are presented across the temperature interval of 25–150 °C. Figure 13 illustrates the temperature–viscosity curves of a 1 mmol/L EUCGS solution and CTAB solution. The analysis reveals that there exists a complex nonlinear relationship between the rheological behavior of the EUCGS solution and temperature, and this relationship is significantly modulated by the shear field intensity. In the low shear region (< 0.1 s^−1^), the apparent viscosity is particularly sensitive to temperature changes. With the temperature elevation from 25 °C to 50 °C, a 44.6% decline in the zero shear viscosity was observed. Upon subsequent heating up to 75 °C, the viscosity value dropped to 21.8% of the initial measurement. It is worth emphasizing that a pivotal transition occurs between 100 and 125 °C. During this temperature span, there is a remarkable 73.4% drop in viscosity, thereby implying a substantial transformation of the internal structural arrangement. In contrast, in the high shear region (>10 s^−1^), the temperature response below 100 °C is much smoother, suggesting that the intermolecular interactions are partially disrupted under high shear conditions, and the temperature effect is correspondingly weakened. The investigation of the high shear region indicates that the viscosity of the solution exhibits a relatively slow decline when the temperature is maintained below 100 °C. Conversely, once the temperature surpasses 125 °C, a drastic decrease in the solution’s viscosity is observed. The experiment demonstrates that the developed EUCGS exhibits excellent temperature resistance at 100 °C.

Figure 13 shows the viscosity of EUCGS and CTAB variations with temperature under different shear rates in comparison with the CTAB system data, revealing that both surfactants exhibit similar temperature-responsive behavior, although the EUCGS demonstrates slightly higher viscosity values at all temperature points. Under low shear conditions at 25 °C, the viscosity of EUCGS is approximately 27.6% higher than that of CTAB, whereas the difference between the two becomes negligible under high shear conditions. This discrepancy is primarily attributed to the more robust entanglement density and network connectivity of the micellar network formed by the gemini molecular structure of EUCGS. Analysis of the high shear region indicates that when the temperature is below 100 °C, the viscosity of both EUCGS and CTAB solutions decreases relatively slowly, dropping from 36 and 30 mPa·s at 25 °C to 30.8 and 16 mPa·s at 100 °C, respectively. When the temperature exceeds 125 °C, the viscosity of both solutions decreases sharply, reaching 12 mPa·s at 150 °C, respectively, and becoming nearly identical. The experimental results demonstrate that the developed EUCGS possesses excellent temperature resistance at 100 °C, with temperature-responsive characteristics similar to those of the CTAB system, but it exhibits a superior viscosity retention capability under low shear conditions.

The unique temperature resistance mechanism of EUCGS originates from its precisely designed molecular architecture. The long hydrocarbon chains containing cis double bonds undergo conformational transitions under thermal stress, enabling the molecules to adopt a more compact arrangement and maintain hydrophobic interactions with sufficient strength [37]. Moreover, the connecting groups in the geminate structure not only serve as spatial constraints but also regulate the balance between hydrophobic interactions and electrostatic repulsion through the intramolecular entropy effect. At the microscopic level, the thermal response of the EUCGS solution involves a complex micellar phase transition process. The temperature-induced morphological transformation of micelles generally follows the evolutionary path of “vermiform → short rod-like → spherical → monomer”. And the decrease in viscosity is well explained by length reduction [38]. In the EUCGS system, this transformation process is significantly regulated by molecular design. In the temperature range of 25–75 °C, the micellar network responds to temperature changes mainly by reducing the number of entanglement points and decreasing the entanglement lifetime, while the tubular structure and rigidity of the micelles remain basically unchanged. In the temperature range of 75–100 °C, the micellar structure begins to change significantly, and the continuous length of the wormlike micelles gradually shortens, but the network structure remains basically intact. When the temperature exceeds 125 °C, the system undergoes a cooperative phase transition, the micellar network structure disintegrates, and it transforms into dispersed spherical or short rod-like micelles, leading to a sharp decline in rheological properties.

The multi-level thermoresponsive properties of EUCGS render it particularly suitable for fracturing environments characterized by significant temperature variations. Under low-temperature conditions at the surface, its high viscosity facilitates proppant transport. During injection into high-temperature reservoirs, the viscosity decreases moderately to reduce pumping resistance. In the temperature gradient zones of reservoir fractures, EUCGS can automatically adjust its rheological properties according to local temperatures, enabling “intelligent” proppant transport and flowback control [39].

### 3.3. Performance Evaluation of Fracturing Fluids

#### 3.3.1. Proppant-Carrying Performance Evaluation

The settling rates of proppants in different fracturing fluid systems at various temperatures are presented in Table 2. Experimental results show that with the increase in temperature, the settling rates of proppants in both fracturing fluid systems exhibit an upward trend, but the values are far lower than the industry standard of 5 cm/min. This is because the increase in temperature reduces the viscosity of the fracturing fluid, weakening its suspending capacity for proppants. However, due to the good temperature stability of both surfactants, the increase in settling rate is relatively controllable.

The developed EUCGS-FFS demonstrates a superior proppant-carrying performance across all temperature ranges compared to the traditional CTAB-FFS fracturing fluid system, which is attributed to the unique C22 ultra-long-chain gemini molecular structure of EUCGS. This structure enables it to form a more stable and dense wormlike micelle network, providing stronger viscoelasticity and better suspending and supporting capabilities. Specifically, under the high-temperature condition of 90 °C, the settling rate of EUCGS-FFS (0.25 cm/min) is still significantly lower than that of CTAB-FFS (0.42 cm/min), reflecting its excellent high-temperature, proppant-carrying performance and application potential in deep well fracturing operations.

#### 3.3.2. Gel-Breaking Performance Evaluation

**Figure 14:**
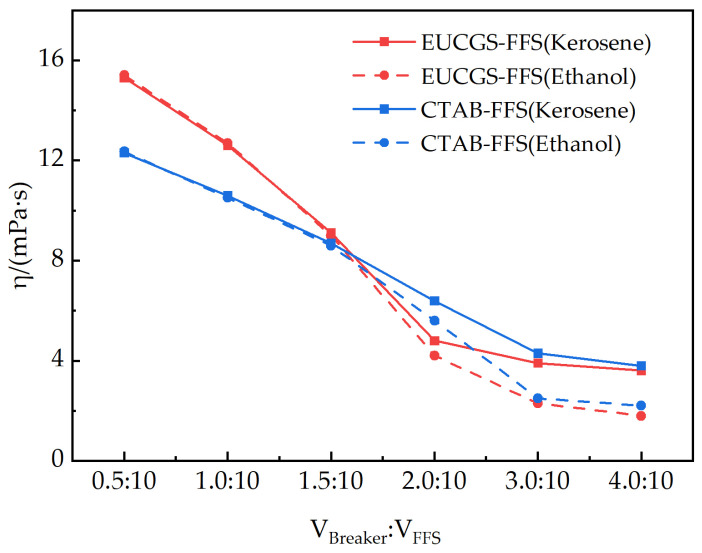
The gel-breaking performance of fracturing fluids under the action of different types and dosages of gel breakers.

For the developed fracturing fluid system EUCGS-FFS and the conventional fracturing fluid system CTAB-FFS, both kerosene and absolute ethanol can serve as gel breakers. The viscosities of the two fracturing fluid systems 2 h after gel breaking under different types and dosages of gel breakers are shown in Figure 14. It can be seen from the figure that with the increase in gel breaker dosage, the viscosities of both fracturing fluid systems show a significant downward trend. Taking 5 mPa·s as the evaluation standard for complete gel breaking, EUCGS-FFS demonstrates a more excellent gel-breaking performance: when the volume ratio of the gel breaker is 2.0:10, the residual viscosities of EUCGS-FFS under the action of kerosene and ethanol are 4.8 and 4.2 mPa·s, respectively, both of which have reached the standard of complete gel breaking. In contrast, the viscosities of CTAB-FFS under the same conditions are still 6.4 and 5.6 mPa·s, respectively, indicating incomplete gel breaking.

The excellent gel-breaking performance of EUCGS-FFS is primarily attributed to its unique molecular structural characteristics. First, the amide bonds in EUCGS molecules provide specific action sites for gel breakers. Polar gel breakers (such as ethanol) can effectively interfere with the intermolecular interactions around amide bonds through hydrogen bonding [40]. Second, the cis double bonds in the erucyl long chain cause the molecular chain to exhibit a bent configuration [41], reducing the degree of ordered arrangement between molecules and making the micellar network more prone to depolymerization under the action of gel breakers. Finally, the connecting group (1,3-propanediol derivative) in the gemini structure contains hydroxyl groups, which enhance the hydrophilicity of molecules and facilitate the penetration and action of gel breakers. When the volume ratio of the gel breaker reaches 3.0:10, the residual viscosity of all systems drops below 3 mPa·s, indicating that both fracturing fluid systems have good controllable gel-breaking properties. However, EUCGS-FFS can achieve rapid gel breaking with a lower gel breaker dosage, which is more in line with the requirements of economical and efficient fracturing operations [42,43,44].

#### 3.3.3. Evaluation of Core Damage Performance

Table 3 presents the experimental results of core permeability for EUCGS-FFS and CTAB-FFS. As shown in the table, the initial permeability (*K*_0_) of the two fracturing fluid systems is basically the same (approximately 36.5 × 10^−3^ μm^2^), but they exhibit different damage characteristics during the injection process. The initial permeability damage rate (Φ) of EUCGS-FFS is 11.8%, slightly higher than 9.3% of CTAB-FFS, which is mainly attributed to the gemini structure and C22 ultra-long-chain of EUCGS molecules, enabling the formed micellar network to be more stable and dense, thus resulting in a relatively higher retention degree in core pores. Specifically, as a gemini surfactant, the EUCGS has a significantly larger molecular size than the single-chain CTAB molecule, making it more susceptible to physical entrapment within the fine pores of rock cores. Meanwhile, the wormlike micellar network formed by EUCGS exhibits enhanced viscoelasticity and network connectivity, resulting in greater fluid resistance at pore throats. Furthermore, the gemini molecular structure provides more adsorption sites, which may lead to the formation of a more stable initial adsorption layer on the rock surface.

However, the EUCGS-FFS demonstrates significant advantages in the subsequent recovery stage. Its permeability recovery rate (*R*) reaches 12.4%, significantly higher than 9.0% of CTAB-FFS, and the final damage rate (Φ’) is only 0.9%, superior to 1.1% of CTAB-FFS. This excellent recovery performance is attributed to the unique molecular structural characteristics of EUCGS: The amide bonds in the molecule and the hydroxyl groups on the connecting groups enhance its water solubility, which is conducive to the dissolution and cleaning of residual substances; the cis-double bonds in the erucyl long chain make the molecules present a bent configuration, reducing the irreversible adsorption on the surface of core pores; and although the gemini structure forms a stable network initially, it can be more thoroughly depolymerized and removed under gel-breaking and flushing conditions. In contrast, although CTAB molecules induce relatively minor initial damage, their residual presence within the core is more challenging to completely eliminate, resulting in a comparatively higher permanent damage rate. In general, although EUCGS-FFS has a slightly higher initial damage rate, it has better reversibility and lower permanent damage, making it more suitable for fracturing operations with high requirements for reservoir protection.

## 4. Conclusions

In this study, a novel erucyl-based ultra-long-chain gemini surfactant (EUCGS) was designed and synthesized, with its structural characteristics and performance systematically evaluated. The key findings are summarized as follows:

(1) Molecular synthesis and characterization: The target product EUCGS was prepared via a two-step synthesis method, achieving an overall yield of 78.6%. FT-IR and ^1^H NMR analyses confirmed the presence of a C_22_ ultra-long chain, cis double bond, amide bond, and quaternary ammonium head group in the molecular structure.
(2) Surface activity: EUCGS exhibited a low critical micelle concentration (0.018 mmol/L) and excellent surface tension reduction capability (γCMC = 30.0 mN/m), with these parameters outperforming those of conventional single-chain surfactants.
(3) Rheological properties: At a concentration of 1.0 mmol/L, EUCGS formed a wormlike micellar network, displaying Maxwell fluid characteristics and shear-thinning behavior. In the presence of 225 mmol/L NaCl, the apparent viscosity of 20 mmol/L EUCGS solution increased from 86 mPa·s to 255 mPa·s. The solution demonstrated good thermal stability at 100 °C.
(4) Fracturing fluid application performance: The proppant settling rate of the EUCGS-FFS system at 90 °C (0.25 cm/min) was lower than that of the CTAB-FFS system (0.42 cm/min). Additionally, the EUCGS-FFS system required a lower dosage of the breaker and resulted in a final core damage rate of 0.9%.

The research results indicate that through rational molecular structure design, EUCGS achieves the coordinated optimization of interfacial activity, rheological properties, and thermal–salt stability, making it a promising candidate material for clean fracturing fluids. Further studies are required to verify its application effectiveness under actual fracturing conditions.

## Figures and Tables

**Table 1:** Comparison of surface activity parameters between EUCGS and typical surfactants at 25 °C.

Surfactants	CMC (mmol/L)	γCMC (mN/m)	Γmax (10^−3^ mmol/L)	Amin (nm^2^)
EUCGS	0.018	30.0	1.28	1.30
JS-N-JS [26]	0.028	33.12	1.59	1.04
VES-Q [15]	0.047	43.2	0.388	2.33
CTAAC [27]	0.15	30.65	3.16	0.28
CTAB [28]	0.94	39	1.25	1.33

**Table 2:** The settlement rate of proppants in different fracturing fluid systems at various temperatures.

Temperature/°C	Sedimentation Rate/(cm·min^−1^)	
	EUCGS-FFS	CTAB-FFS
30	0.0001	0.0001
70	0.13	0.23
90	0.25	0.42

**Table 3:** The evaluation of core permeability for different fracturing fluid systems.

	*K*_0_/10^−3^ μm^2^	*K*_1_/10^−3^ μm^2^	*K*_2_/10^−3^ μm^2^	Φ/%	*R*/%	Φ’/%
EUCGS-FFS	36.45	32.15	36.14	11.8	12.4	0.9
CTAB-FFS	36.52	33.14	36.12	9.3	9.0	1.1

## Data Availability

The original contributions presented in this study are included in the article material. Further inquiries can be directed to the corresponding author.
